# Dental fear association between mothers and adolescents—a longitudinal study

**DOI:** 10.7717/peerj.9154

**Published:** 2020-05-13

**Authors:** Hai Ming Wong, Yu Yuan Zhang, Antonio Perfecto, Colman P.J. McGrath

**Affiliations:** 1 Paediatric Dentistry & Orthodontics, Faculty of Dentistry, The University of Hong Kong, Hong Kong SAR, China; 2 Dental Public Health, Faculty of Dentistry, The University of Hong Kong, Hong Kong SAR, China

**Keywords:** Dental fear, Oral preventative health, Modified dental anxiety scale, Dental anxiety, Adolescent oral health

## Abstract

**Aim:**

To assess the longitudinal association between adolescents’ and their mothers’ dental fear.

**Study Design:**

A longitudinal questionnaire survey study.

**Methods:**

A randomized sample of 12-year-old adolescents were selected from local Hong Kong schools. Adolescents and their mothers self-completed the Modified Dental Anxiety Scale (MDAS). The sociodemographic background of the mothers and the oral health habits of the adolescents were also collected and these measurements were repeated at 15- and 18-years-old. Non-parametric tests (Mann–Whitney *U* test/Kruskall Wallis test) were used to test associations between MDAS dental fear items and independent variables. Logistic regression (adjusted for family’s sociodemographic background and adolescent’s oral health habits) was performed to evaluate the longitudinal association between adolescents’ and mothers’ dental fear.

**Results:**

A total of 212 mother-child pairs were recruited at baseline (12-year-old adolescents). In the first and second follow-ups (15- and 18-years-old), 195 and 182 mother-child pairs completed the survey. Significant associations between mother’s and child’s scores in “feeling about having their teeth scraped and polished”, “having teeth drilled”, and ‘having an injection in the gum’ were found when adolescents were 12- years-old (*P* < 0.01) and 18-years-old (*P* < 0.05), but not at 15-years-old.

**Conclusion:**

Adolescents’ and mothers’ dental fear is associated at 12-years-old and 18-years-old, but not at 15-years-old, which is likely specific to the Hong Kong context but may be extrapolated to other industrialized countries with caution.

## Introduction

In recent years, there has been a growing interest in underpinning the psychological barriers involved in oral preventative health. One such psychological barrier is dental fear, causing either the delay or absence of dental visitation, which is detrimental to the utilization of health services, oral health outcomes, and health care-related costs. Dental fear comprises all age groups but one of the most vulnerable groups, in particular, are children and adolescents. The global prevalence of dental fear in children and adolescents ranges between 10% and 20% (Klingberg & Broberg, 2007; Cianetti et al., 2017) with studies demonstrating that lower dental fear within this population is associated with improved oral health status (Klaassen, Veerkamp & Hoogstraten, 2003).

Addressing dental fear in children is a cost-effective strategy that may help to pre-empt deleterious dental outcomes, such as cavities and periodontitis. Several contributing factors elicit dental fear in children and adolescents. For example, gender has a strong influence in shaping dental fear in adolescents and children as dental fear disproportionally affects women, irrespective of age. Women have higher rates of dental anxiety compared to men (Armfield, Slade & Spencer, 2008), which has been partly attributed to, based on self-reported surveys, increased fearfulness (Armfield, Spencer & Stewart, 2006). This is particularly evident in maternal dental anxiety, which is demonstrated to result in poorer oral health outcomes in children (Goettems et al., 2011). In speculation regarding the mechanism behind poorer oral health outcomes, one potential hypothesis is that maternal dental anxiety directly increases adolescent dental fear and, in turn, is a major cause of poor oral health. If this were the case, these findings would have important implications in childhood oral health.

In general, the results from previous studies that have investigated whether a relationship between maternal and child dental anxiety exists has been mixed. Veerkamp et al. (1992) showed that parents with high levels of dental anxiety projected their feelings onto their children and negatively influenced their child’s behaviors toward dental visitation. In contrast, other studies have not found significant correlations between dental anxiety and parental rearing styles (Krikken et al., 2013). It is important to note that other factors unrelated to parental anxiety can also influence children’s dental fear. For instance, family income affects children’s dental anxiety levels (Oliveira & Colares, 2009) and adolescents from lower income families tend to have a higher probability of dental anxiety (Assunção et al., 2013). In addition, some researchers have speculated that the child’s dental anxiety actually originates from the generalized psychological status of the parent as opposed to the dental anxiety of the parent (Marsac & Funk, 2008). Given all of the evidence, the etiology of dental fear is both multi-factorial and multi-dimensional (Klingberg & Broberg, 2007; Locker, Thomson & Poulton, 2001; Wong, Mak & Xu, 2011).

Maternal dental anxiety, fear, and its potential influence in adolescent dental fear is likely a result of parental pre-conceived dispositions towards dental treatment. Previous studies have shown that parents have a considerable influence in childhood-onset anxiety that results in dental fear (Locker et al., 1999). Inferred from the study is that parental unintentional behaviors, such as negative feelings regarding dental procedures, shape children and adolescents perceptions and behaviors at a young and vulnerable stage. To date, parental education level is shown to be inversely correlated with general anxiety levels in adolescents (Stenebrand, Wide Boman & Hakeberg, 2013) and dental anxiety levels of children (Cardoso, Loureiro & Nelson-Filho, 2004). Longitudinal data informing the relationship between maternal dental fear and adolescent dental fear is currently lacking.

Given the importance of adolescent oral health (Li et al., 2015; Sun, Wong & McGrath, 2015) and the lack of data regarding the influence of maternal dental fear on adolescent dental fear, the objective of this study was to evaluate the longitudinal impact of mothers dental fear on their children’s dental fear from 12- to 18-years-old after adjusting for family’s sociodemographic background and adolescent’s oral health habits.

## Materials and Methods

### Sample size calculation

In order to calculate the sample size, the estimated standard deviation (SD) was derived from a previous study (Wong, Humphris & Lee, 1998). A total of 150 mother-child pairs were required for 85% power to identify a significant difference using paired *t*-test/Wilcoxon matched pair test at 5% level of significance (alpha = 0.05). The power analysis was conducted using the G*Power 3.1.9.2 software (http://www.psycho.uni-duesseldorf.de/abteilungen/aap/gpower3). Given an anticipated dropout rate of 20%, a minimum of 180 patients were required for recruitment.

### Sample

For the initial baseline survey, participants were drawn from local secondary schools based in Hong Kong. Five schools were chosen at random (1% of all local secondary schools). All 12-year-old adolescents were invited to participate in the survey within each school. The exclusion criteria included students not of southern Chinese ethnicity and/or afflicted with systemic diseases with clear connections to oral health, such as Type-2 diabetes mellitus, cardiovascular disease, and immunocompromised individuals. The first follow-up survey was conducted when participants were 15-years-old, and the second follow-up survey when participants were 18-years-old.

### Data collection

Mothers and their adolescents were given two separate but identical questionnaires to complete in each survey. The first questionnaire was composed of questions related to family sociodemographic background and the oral health habits for adolescents. The second questionnaire was based off the self-reported Modified Dental Anxiety Scale (MDAS) (Humphris, Morrison & Lindsay, 1995). The MDAS questionnaire consisted of five questions designed to assess the participant’s feelings in five different dental situations. The MDAS used a scale of 1–5, from not anxious to extremely anxious, with total scores ranging from 5 to 25.

### Statistical methods

Mean, SD and median were used to describe continuous data. Non-parametric tests (related-samples Friedman’s two-way analysis of variance by ranks) compared MDAS scores amongst 12-, 15-, and 18-year-olds.

For each period of data collection, associations of multiple dental fear items and independent variables (employment status, family income, maternal education level, frequency of tooth brushing, frequency of snack intake, etc.) were analyzed using non-parametric tests (Mann–Whitney *U* test/Kruskall Wallis test).

Logistic regression was used to evaluate the longitudinal association between mother and adolescent’s dental fear. In the regression model, gender, socioeconomic factors (employment status, family income, mother’s education, father’s education) and behavioral factors (frequency of tooth brushing, use of fluoride toothpaste, frequency of snacking) were adjusted. All statistical analyses were conducted using SPSS 23.0. The level of statistical significance of 0.05 was set for all analyses.

### Ethical considerations

This study was approved by the Institutional Review Board of the University of Hong Kong (IRB reference number: UW 15-278). Mothers and adolescents were informed of the details regarding the study, and their participation was completely voluntary. Written consent was obtained from the mothers and on behalf of their children at 12-years-old prior to enrollment and was re-obtained when adolescents were 18-years-old.

## Results

A total of 212 mother-child pairs were recruited at baseline. In the first follow-up, 195 15-year-olds participated in the survey (8% drop out rate) and in the second follow-up, 182 18-year-olds participated (6.7% drop out rate). The sole reason given for withdrawal was school transfer. Of the remaining 182 adolescents, the ratio of male to female was 110:72.

Adolescents dental fear scores of “feeling about sitting in the waiting area” (*P* < 0.001) and “having their teeth scraped and polished” (*P* < 0.01) were significantly higher at 12-years-old than at 18-years-old. Mothers dental fear scores of “drilled” (*P* < 0.05) and “injection” (*P* < 0.01) were significantly higher when their children were 12-years-old compared to 15-years-old (*P* < 0.05). However, the inverse relationship was observed for “feeling about seeing a dentist tomorrow”, as mothers dental fear scores were significantly higher when their children were 15-years-old as compared to 12-years-old (Tables 1 and 2).

**Table 1:** Description of mother’s and 12-, 15-, and 18-years-old adolescent’s dental fear.

Variable	12 years/old (1)	15 years/old (2)	18 years/old (3)	*P*-value	Multiple comparison
	Q1/Median/Q3	Q1/Median/Q3	Q1/Median/Q3		
Child’s dental fear					
How do you feel if you have to see a dentist tomorrow?	1/2/3	1/2/3	1/1/2	NS	(1) = (2) =(3)
How do you feel about sitting in the waiting area?	1/2/3	1/2/3	1/1/2	<0.0001***	(1) = (2); (2) = (3); (1) > (3)
How do you feel about having your teeth drilled?	2/3/4	2/3/4	2/3/4	NS	(1) = (2) = (3)
How do you feel about having your teeth scraped and polished?	1/2/3	1/2/3	1/2/3	0.004**	(1)= (2); (2) = (3); (1) > (3)
How do you feel about having an injection in the gum?	2/3/4	2/3/4	2/3/4	NS	(1) = (2) = (3)
MDAS	7/11/15	7/11/15	6/10/14	NS	(1) = (2) = (3)
Mother’s dental fear					
How do you feel if you have to see a dentist tomorrow?	1/1/2	1/2/3	1/2/3	0.017*	(2) > (1); (1) = (3); (2) = (3)
How do you feel about sitting in the waiting area?	1/2/3	1/2/3	1/2/3	NS	(1) = (2) = (3)
How do you feel about having your teeth drilled?	2/3/4	2/3/4	2/3/4	0.015*	(1) > (2); (2) = (3); (1) = (3)
How do you feel about having your teeth scraped and polished?	1/2/3	1/2/3	1/2/3	NS	(1) = (2) = (3)
How do you feel about having an injection in the gum?	2/3/4	1/2/3	2/3/4	0.001**	(1) > (2); (1) = (3); (2) = (3)
MDAS	9/12/15	7/10/14	7/11/15	NS	(1) = (2) = (3)

**Notes:**

MDAS: The Modified Dental Anxiety Scale.

Non-parametric tests, related-samples Friedman’s two-way analysis of variance by ranks were used by multiple comparison.

* *P* < 0.05.

** *P* < 0.01.

*** *P* < 0.001.

**Table 2:** Socio-demographic variations in mother’s dental fear when adolescents were 12-, 15-, and 18-years-old.

Variable	*n* (years/old) 12/15/18	MDAS (12 years/old)		MDAS (15 years/old)		MDAS (18 years/old)	
		Q1/Median/Q3	*P* value	Q1/Median/Q3	*P* value	Q1/Median/Q3	*P* value
Parent’s employment status			NS		NS		NS
Both employed	108/115/110	7/11/15		7/11/15		7/11/15	
At least one unemployed	72/65/70	7/11/16		6/10/14		6/11/16	
Family income			NS		NS		NS
Less than HK$ 10,000	44/27/21	7/11/15		6/10/14		7/11/15	
HK$ 10,001–HK$ 30,000	95/101/97	6/11/15		7/11/15		6/11/15	
More than HK$ 30,000	38/52/60	7/11/15		7/11/15		7/11/14	
Education			NS		NS		NS
Junior High School or below	79/79/78	5/10/15		6/10/14		6/10/14	
High School	81/80/81	4/11/14		7/11/15		6/11/16	
University or above	21/21/21	9/12/15		8/11/14		9/12/15	

**Notes:**

MDAS: The Modified Dental Anxiety Scale.

Dental visit: How do you feel if you have to see a dentist tomorrow?

Waiting: How do you feel about sitting in the waiting area?

Drilling: How do you feel about having your teeth drilled?

Scaling: How do you feel about having your teeth scraped and polished?

Injection: How do you feel about having an injection in the gum?

*P* values were calculated in non-parametric tests, Mann–Whitney *U* test/Kruskall Wallis test.

Mothers dental fear scores for “feeling about having an injection in the gum” were significantly higher when adolescents were 15-years-old and when family income was more than 30,000 HKD per month (*P* < 0.05) (Table 2). The same dental fear score for ‘injection’ was significantly higher when adolescents were 18-years-old and when mothers held a University education or above (*P* < 0.05). Similarly, mothers dental fear scores for “drilled” were significantly higher in 18-year-olds with maternal higher education (*P* < 0.05) (Table 2). Significant differences in several dental fear scores between males and females were observed for 15- and 18-years-old adolescents, but not at 12-years-old (Table 3). Amongst all oral-health behavioral variations, only “frequency of taking snack” was significantly associated with the fear of “drilling” at 12-years-old (Appendix 5).

**Table 3:** Socio-demographic and oral-health behavioral variations in 12-, 15-, and 18-year-old adolescents’ dental fear.

Variable	*n* (years/old) 12/15/18	MDAS (12 years/old)		MDAS (15 years/old)		MDAS (18 years/old)	
		Q1/Median/Q3	*P* value	Q1/Median/Q3	*P* value	Q1/Median/Q3	*P* value
Gender			NS		0.001**		0.001**
Male	109/110/110	6/11/15		6/11/16		7/11/15	
Female	71/72/72	7/11/15		6/9/12		5/9/13	
Parent’s employment status			NS		NS		NS
Both employed	108/115/110	6/11/16		7/11/15		6/10/15	
At least one unemployed	70/66/70	7/11/15		7/11/16		5/10/15	
Family income			NS		NS		NS
Less than HK$ 10,000	44/27/21	8/12/16		4/9/14		6/10/14	
HK$ 10,001–HK$ 30,000	94/101/97	7/11/15		7/11/15		6/10/15	
More than HK$ 30,000	37/52/60	6/11/16		7/11/15		6/10/14	
Mother’s education			NS		NS		NS
Junior High School or below	78/79/79	8/12/16		7/11/15		6/10/14	
High School	80/81/81	6/10/14		6/10/14		7/10/14	
University or above	21/21/21	7/12/17		7/11/15		7/11/15	
Father’s education			NS		NS		NS
Junior High School or below	91/91/91	8/12/16		7/11/16		7/10/14	
High School	57/57/57	6/10/14		7/11/15		6/10/14	
University or above	30/30/30	5/10/15		6/11/15		7/11/15	
Frequency of tooth brushing			NS		NS		NS
Less than twice a day	39/40/43	8/12/16		7/11/16		6/10/15	
At least twice a day	143/141/139	7/11/15		7/11/15		6/10/14	
Use of fluoride toothpaste			NS		NS		NS
Yes	68/93/104	6/10/14		6/10/14		6/10/14	
No or not sure	112/89/74	7/12/15		7/11/15		7/10/14	
Frequency of taking snack			NS		NS		NS
Less than once a day	39/36/44	8/13/16		7/11/15		6/10/14	
Once a day	98/93/82	6/10/14		6/11/15		6/10/15	
At least twice a day	43/53/56	7/12/16		7/11/15		7/11/15	

**Notes:**

MDAS: The Modified Dental Anxiety Scale.

Dental visit: How do you feel if you have to see a dentist tomorrow?

Waiting: How do you feel about sitting in the waiting area?

Drilling: How do you feel about having your teeth drilled?

Scaling: How do you feel about having your teeth scraped and polished?

Injection: How do you feel about having an injection in the gum?

*P* values were calculated in non-parametric tests, Mann–Whitney *U* test/Kruskall Wallis test.

** *P* < 0.01.

Significant associations were found between mother and child’s scores in “feeling about having their teeth scraped and polished”, “having teeth drilled”, and “having an injection in the gum”, at 12-years old (*P* < 0.01) and 18-years old (*P* < 0.05) (Table 4). At 15-years-old, no associations were observed between mother and child’s dental fear scores.

**Table 4:** Association between mother’s and adolescent’s dental fear at 12-, 15-, and 18-years-old.

Independent variable	12-years-old			15-years-old			18-years-old		
	Estimate	95% CI	*P*	Estimate	95% CI	*P*	Estimate	95% CI	*P*
Dental visit	1.66	[0.57, 4.79]	NS	1.42	[0.45, 4.53]	NS	1.14	[0.21, 6.12]	NS
Waiting	1.66	[0.57, 4.91]	NS	0.31	[0.07, 1.46]	NS	1.66	[0.60, 4.63]	NS
Scaling	3.72	[1.68, 8.20]	0.001**	2.19	[0.87, 5.51]	NS	2.84	[1.21, 6.71]	0.017*
Drilling	3.01	[1.51, 5.99]	0.002**	1.29	[0.69, 2.40]	NS	2.80	[1.48, 5.31]	0.002*
Injection	4.56	[2.22, 9.39]	<0.001**	1.28	[0.64, 2.58]	NS	2.43	[1.28, 4.60]	0.007*
MDAS	6.01	[0.48, 75.48]	NS	−0.753	[−1.96, 0.088]	NS	7.31	[0.99, 53.73]	NS

**Notes:**

MDAS: The Modified Dental Anxiety Scale.

Dependent variable (continuous data): Dental fear of children.

Independent variable (categorical data): Dental fear of mothers.

Dental visit: How do you feel if you have to see a dentist tomorrow?

Waiting: How do you feel about sitting in the waiting area?

Drilling: How do you feel about having your teeth drilled?

Scaling: How do you feel about having your teeth scraped and polished?

Injection: How do you feel about having an injection in the gum?

The model was assessed by logistic regression; adjusted for gender (categorical data), socioeconomic factors (parents’ employment; family income; mother’s education; father’s education) (categorical data) and behavioral factors in 12-year-old (frequency of tooth brushing, use of fluoride toothpaste and frequency of snacks) (categorical data).

* *P* < 0.05.

** *P* < 0.01.

## Discussion

This study investigated the association of dental fear between mothers and adolescents from early to late stage adolescence (12-, 15-, 18-years old) amongst a population-representative sample in Hong Kong. Our study attempted to reveal the longitudinal trends of an association during a critical developmental and cognitive phase in children.

One of the strengths of our study is that we provided one questionnaire that was used by both mothers and their children. In previous research studies using survey questionnaires that have attempted to uncover correlations of emotionality between the parent and child, entirely different questionnaires were targeted toward either the parent or the child (Themessl-Huber et al., 2010). To our knowledge, the use of different questionnaires has not been validated for use in both parents and their children and may contribute to the inconsistent findings regarding whether parental dental anxiety is associated with their children’s dental fear (Leal et al., 2013). The questionnaire in our study was based off a previously validated survey that was appropriate for use by parents and children and specifically measures dental anxiety. An advantage of the validated child dental anxiety scale (MDAS) (Wong, Humphris & Lee, 1998) is it is highly adaptable; the survey can be modified according to the specific research hypotheses and/or objectives. Other advantages for using the MDAS include evidence for reliability, quick completion, wide spread use in experimental and clinical studies, and the ability to compare its results with other published studies (Corah, Gale & Illig, 1978).

Our adolescent cohort that originally enrolled in our study at 12-years-old reported no difficulties in comprehension in response to the MDAS. Given that both mothers and their adolescents completed the same MDAS survey, we are confident in the appropriateness of our findings and interpretations comparing mothers dental anxiety to their adolescents dental anxiety.

Moreover, aside from two distinct surveys given to the mother and the child, the variability in survey design is also evident in previous studies. Interestingly, studies not using validated surveys report significant relationships between parental and child dental fear more often than studies using validated surveys (Themessl-Huber et al., 2010). Although one study found that parent and adolescent dental fear was independent of one another in 11–12 and 15–16 year olds, they utilized single-item questionnaires (Luoto et al., 2017), which are not as robust as multi-item question scales capturing different aspects of dental fear (Armfield, 2010). Indeed, Themessl-Huber et al. (2010) recommended in a systematic review that comprehensive and validated measurement scales to assess dental fear in parents and children were required for valid and reliable research outcomes, providing further justification for the use of the MDAS.

Unique to our study is that we investigated the role of mother and child dental anxiety with a longitudinal approach. An age-dependent effect on the relationship between parental and child dental fear has not been fully elucidated. Themessl-Huber et al. (2010) also showed that children’s dental fear under the age of eight was significantly associated with parental dental fear, which suggests that an earlier age may especially be prone to a mother’s dental fear. One explanation may be the greater effect of parental influence on younger children (Assunção et al., 2013). Another plausible reason may be that children at an earlier age are likely to have had fewer or no prior dental experiences and their parents frame their perception of dental visitation. As children age, they have a better understanding of dental visitation, develop coping mechanisms, and gradually accept things all due to habituation which we assumed would disrupt the degree of influence from the mother’s dental fear. Based on this premise, we anticipated that our results would reflect a trend of decreasing association mothers dental fear on their adolescents dental fear over time. This was partly the case in our study as significant associations were found between mother and child’s MDAS scores “in feeling about having their teeth scraped and polished”, “having teeth drilled”, and “having an injection in the gum” at 12-years-old (*P* < 0.01) but not at 15-years-old. At 15-years-old, it is possible that a higher degree of autonomy results in less influence from the mother, and subsequently less dental fear. Based on this reasoning, we unexpectedly found that the association was significant at 18-years old, suggesting there may be other factors related to the child’s dental fear that that we were unable to account for. This is an interesting finding, as it would infer that other factors override age-dependent autonomy in the association of dental fear between mothers and adolescents.

Our results are not entirely without precedent as a previous study showed accelerated rates of dental fear with advancing age. When separated categorically by age, the incidence of dental fear amongst 40–60 year-olds was 22%, 25–39 year-olds (17%), and 18–24 year-olds (13%) (Armfield, Spencer & Stewart, 2006). There is a possibility that higher levels of socialization and cognitive ability with increasing age perpetuates psychological and emotional distress resulting in high dental anxiety (Armfield, Slade & Spencer, 2008). Therefore, we may have observed an inflection point trending toward dental fear with our cohort at 18-years-old. We also speculate that within the Hong Kong school context, the association of dental anxiety between mothers and children may be caused by stress levels induced within the academic system. At 18-years-old, Hong Kong adolescents undergo a highly competitive Diploma of Secondary Education Examination, which largely determines their university admissions. A novel finding of our study was that “frequency of taking snack” was significantly associated with the fear of “drilling”. Given that dental anxiety predicts caries incidence in adolescence, and frequency of snacking is strongly associated with caries (Kruger et al., 1998), the association of “snacking” and its association with “drilling” is likely mediated by caries.

In our study, we also found that at least for one dental fear score, an increase in mothers dental anxiety was associated with higher income and higher education, which was unanticipated given previous findings (Oliveira & Colares, 2009; Assunção et al., 2013). Besides the direct effects of mothers’ dental fear, the father’s dental fear and possibly other socioeconomic factors are likely to have a strong influence on adolescents’ dental fear. Investigating the role of the father in dental fear and understanding the role of socioeconomic factors, and more broadly the societal effects on dental anxiety in Hong Kong could be explored in future studies.

## Conclusions

Adolescents dental fear was associated with his/her mother’s dental fear at 12- and 18-, but not at 15-years-old. Our results are likely context dependent, based upon the unique aspects of our Hong Kong cohort. Thus, the association of dental anxiety between mothers and adolescents remains not fully understood and requires accounting for confounding factors. Additional longitudinal studies using valid and reliable measurements are required to understand the strength of the association. This would assist in guiding appropriate strategies to target the most vulnerable age-specific adolescents susceptible to dental anxiety.

## Supplemental Information

10.7717/peerj.9154/supp-1 Supplemental Information 1 Socio-demographic variations in mother’s dental fear when adolescents were 12-years-old.

10.7717/peerj.9154/supp-2 Supplemental Information 2 Socio-demographic variations in mother’s dental fear when adolescents were 15-years-old.

10.7717/peerj.9154/supp-3 Supplemental Information 3 Socio-demographic variations in mother’s dental fear when adolescents were 18-years-old.

10.7717/peerj.9154/supp-4 Supplemental Information 4 Socio-demographic and oral-health behavioral variations in 12-year-old adolescents’ dental fear.

10.7717/peerj.9154/supp-5 Supplemental Information 5 Socio-demographic and oral-health behavioral variations in 15-year-old adolescents’ dental fear.

10.7717/peerj.9154/supp-6 Supplemental Information 6 Socio-demographic and oral-health behavioral variations in 18-year-old adolescents’ dental fear.

10.7717/peerj.9154/supp-7 Supplemental Information 7 Raw data. Original dental fear scores

## Additional Information and Declarations

### Data Availability

The following information was supplied regarding data availability: The raw measurements are available in the Supplemental Files.
