# Supplementary material for: Dental fear association between mothers and adolescents—a longitudinal study
Source: PeerJ. 2020 May 13;8:e9154. doi: 10.7717/peerj.9154 (PMC7229765; doi:10.7717/peerj.9154)
Supplement: Supplemental Information 2 [file peerj-08-9154-s002.docx]

**Appendix 2. Socio-demographic variations in mother’s dental fear when adolescents were 15-years-old.**

| **Variable** | **n** | **Dental visit** | | **Waiting** | | **Drilling** | | **Scaling** | | **Injection** | |
| --- | --- | --- | --- | --- | --- | --- | --- | --- | --- | --- | --- |
|  |  | **Q1**  **Median**  **Q3** | ***P* value** | **Q1**  **Median**  **Q3** | ***P* value** | **Q1**  **Median**  **Q3** | ***P* value** | **Q1**  **Median**  **Q3** | ***P* value** | **Q1**  **Median**  **Q3** | ***P***  **value** |
| **Parent’s employment status** |  |  | *NS* |  | *NS* |  | *NS* |  | *NS* |  | *NS* |
| Both employed | 115 | 1  2  3 |  | 1  2  3 |  | 2  3  4 |  | 1  2  3 |  | 2  3  4 |  |
| At least one unemployed | 65 | 1  2  3 |  | 1  2  3 |  | 1  2  3 |  | 1  2  3 |  | 1  2  3 |  |
| **Family income** |  |  | *NS* |  | *NS* |  | *NS* |  | *NS* |  | 0.032* |
| Less than HK$ 10,000 | 27 | 1  2  3 |  | 1  2  3 |  | 1  2  3 |  | 1  2  3 |  | 1  2  3 |  |
| HK$10,001-HK$30,000 | 101 | 1  2  3 |  | 1  2  3 |  | 2  3  4 |  | 1  2  3 |  | 1  2  3 |  |
| More than HK$ 30,000 | 52 | 1  2  3 |  | 1  2  3 |  | 1  2  3 |  | 1  2  3 |  | 2  3  4 |  |
| **Education** |  |  | *NS* |  | *NS* |  | *NS* |  | *NS* |  | *NS* |
| Junior High School or below | 79 | 1  2  3 |  | 1  2  3 |  | 1  2  3 |  | 1  2  3 |  | 1  2  3 |  |
|  |  |  |  |  |  |  |  |  |  |  |  |
| High School | 80 | 1  2  3 |  | 1  2  3 |  | 2  3  4 |  | 1  2  3 |  | 2  3  4 |  |
| University or above | 21 | 1  2  3 |  | 1  2  3 |  | 1  2  3 |  | 1  2  3 |  | 2  3  4 |  |

**Abbreviations:**

Dental visit: How do you feel if you have to see a dentist tomorrow?

Waiting: How do you feel about sitting in the waiting area?

Drilling: How do you feel about having your teeth drilled?

Scaling: How do you feel about having your teeth scraped and polished?

Injection: How do you feel about having an injection in the gum?

MDAS: Modified Dental Anxiety Scale

P: *P < 0.05.

***P* values were calculated in non-parametric tests, Mann-Whitney U test/ Kruskall Wallis test**
