# Supplementary material for: Dental fear association between mothers and adolescents—a longitudinal study
Source: PeerJ. 2020 May 13;8:e9154. doi: 10.7717/peerj.9154 (PMC7229765; doi:10.7717/peerj.9154)
Supplement: Supplemental Information 6 [file peerj-08-9154-s006.docx]

**Appendix 6. Socio-demographic and oral-health behavioral variations in 18-year-old adolescents’ dental fear.**

| **Variable** | **n** | **Dental visit** | | | **Waiting** | | **Drilling** | | **Scaling** | | **Injection** | |
| --- | --- | --- | --- | --- | --- | --- | --- | --- | --- | --- | --- | --- |
|  |  | **Q1 Median**  **Q3** | ***P* value** | **Q1 Median**  **Q3** | | ***P* value** | **Q1 Median**  **Q3** | ***P* value** | **Q1 Median**  **Q3** | **P value** | **Q1 Median**  **Q3** | **P value** |
| **Gender** |  |  | *NS* |  | | 0.025* |  | <0.0001*** |  | 0.001** |  | 0.005** |
| Male | 110 | 1  2  2 |  | 1  2  3 | |  | 2  3  4 |  | 1  2  3 |  | 2  3  4 |  |
| Female | 72 | 1  1  2 |  | 1  1  2 | |  | 1  2  3 |  | 1  1  2 |  | 1  2  3 |  |
| **Parent’s employment status** |  |  | *NS* |  | | *NS* |  | *NS* |  | *NS* |  | *NS* |
| Both employed | 110 | 1  1  2 |  | 1  1  2 | |  | 2  3  4 |  | 1  2  3 |  | 2  3  4 |  |
| At least one unemployed | 70 | 1  1  2 |  | 1  1  2 | |  | 2  3  4 |  | 1  1  2 |  | 2  3  4 |  |
| **Family income** |  |  | *NS* |  | | *NS* |  | *NS* |  | *NS* |  | *NS* |
| Less than HK$ 10,000 | 21 | 1  2  3 |  | 1  1  2 | |  | 1  2  3 |  | 1  2  3 |  | 2  3  4 |  |
| HK$10,001-HK$30,000 | 97 | 1  1  2 |  | 1  1  2 | |  | 2  3  4 |  | 1  1  2 |  | 2  3  4 |  |
|  |  |  |  |  | |  |  |  |  |  |  |  |
| More than HK$ 30,000 | 60 | 1  2  3 |  | 1  1  2 | |  | 2  3  4 |  | 1  2  3 |  | 2  3  4 |  |

| **Mother’s Education** |  |  | *NS* |  | *NS* |  | 0.026* |  | *NS* |  | *NS* |
| --- | --- | --- | --- | --- | --- | --- | --- | --- | --- | --- | --- |
| Junior High School or below | 79 | 1  1  2 |  | 1  1  2 |  | 2  3  4 |  | 1  1  2 |  | 2  3  4 |  |
| High School | 81 | 1  1  2 |  | 1  1  2 |  | 2  3  4 |  | 1  2  3 |  | 2  3  4 |  |
| University or above | 21 | 1  2  3 |  | 1  2  3 |  | 2  3  4 |  | 1  2  3 |  | 2  3  4 |  |
| **Father’s Education** |  |  | *NS* |  | *NS* |  | *NS* |  | *NS* |  | *NS* |
| Junior High School or below | 91 | 1  1  2 |  | 1  1  2 |  | 2  3  4 |  | 1  2  3 |  | 2  3  4 |  |
| High School | 57 | 1  1  2 |  | 1  1  2 |  | 1  2  3 |  | 1  2  3 |  | 2  3  4 |  |
| University or above | 30 | 1  2  3 |  | 1  1  2 |  | 2  3  4 |  | 1  2  3 |  | 2  3  4 |  |

| **Frequency of tooth brushing** |  |  | *NS* |  | *NS* |  | *NS* |  | *NS* |  | *NS* |
| --- | --- | --- | --- | --- | --- | --- | --- | --- | --- | --- | --- |
| Less than twice a day | 43 | 1  1  2 |  | 1  1  2 |  | 2  3  4 |  | 1  2  3 |  | 2  3  4 |  |
| At least twice a day | 139 | 1  1  2 |  | 1  1  2 |  | 2  3  4 |  | 1  1  2 |  | 2  3  4 |  |
| **Use of fluoride toothpaste** |  |  | *NS* |  | *NS* |  | *NS* |  | *NS* |  | *NS* |
| Yes | 104 | 1  1  2 |  | 1  1  2 |  | 1  2  3 |  | 1  2  3 |  | 2  3  4 |  |
| No or not sure | 74 | 1  1  2 |  | 1  1  2 |  | 1  1  2 |  | 1  1  2 |  | 2  3  4 |  |
| **Frequency of taking snack** |  |  | *NS* |  | *NS* |  | *NS* |  | *NS* |  | *NS* |
| Less than once a day | 44 | 1  1  2 |  | 1  1  2 |  | 2  3  4 |  | 1  2  3 |  | 2  3  4 |  |
| Once a day | 82 | 1  1  2 |  | 1  1  2 |  | 1  2  3 |  | 1  1  2 |  | 2  3  4 |  |
| At least twice a day | 56 | 1  2  3 |  | 1  1  2 |  | 2  3  4 |  | 1  2  3 |  | 2  3  4 |  |

**Abbreviations:**

Dental visit: How do you feel if you have to see a dentist tomorrow?

Waiting: How do you feel about sitting in the waiting area?

Drilling: How do you feel about having your teeth drilled?

Scaling: How do you feel about having your teeth scraped and polished?

Injection: How do you feel about having an injection in the gum?

MDAS: The Modified Dental Anxiety Scale

*P*: **P* < 0.05, ***P* < 0.01, ****P*<0.001.

***P* values were calculated in non-parametric tests, Mann-Whitney U test/ Kruskall Wallis test**
